# PKQuest: PBPK modeling of highly lipid soluble and extracellular solutes

**DOI:** 10.5599/admet.579

**Published:** 2018-11-27

**Authors:** David G. Levitt

**Affiliations:** Department of Integrative Biology and Physiology, University of Minnesota, 6-125 Jackson Hall, 321 Church St. S. E., Minneapolis, MN 55455, USA

**Keywords:** pharmacokinetics, anesthetics, interstitial, extracellular, adipose

## Abstract

One of the primary objectives of physiologically based pharmacokinetics (PBPK) is the prediction of a drug’s pharmacokinetics just from knowledge of its physicochemical structure. Unfortunately, at present, the accuracy of this prediction is limited for most drugs because of uncertainty about the drug’s organ/blood partition coefficient (K). However, there are two classes of solutes which are exceptions to this: 1) the highly lipid soluble (HLS) solutes, and 2) the extracellular (ECS) solutes. Since the HLS drugs (eg, volatile anesthetics, propofol, cannabinol) have lipid/water partition coefficients (P_L/W_) of 100 or greater, their K is dominated by the tissue fat fraction and one can accurately predict K just from in vitro measurements of P_L/W_ along with prior anatomic measurements of the fat fraction of the organs in the PBPK model. Since the ECS drugs, such as most antibiotics, cannot penetrate cells, they are not subject to the intracellular binding that complicates the prediction of K for the weak bases and acids. The ECS K is determined primarily by plasma and interstitial albumin binding and can be predicted from in vitro measurements of plasma albumin binding along with prior measurements of interstitial tissue volume and albumin concentrations. This review provides an in depth discussion of the PBPK modeling of these two drug classes along with many specific clinical examples illustrating the good PBPK predictions possible with just zero (volatile anesthetics) or 1 (the clearance) adjustable parameter. The PBPK analysis uses PKQuest, a freely distributed, general purpose pharmacokinetic program. PKQuest is designed so that application to the HLS and ECS solute classes is especially easy. The user only needs to enter the specific parameters that are required to characterize the drug (eg, P_L/W_ for HLS or plasma albumin binding for ECS) with all the other PBPK parameters (organ blood flow, fat fraction, extracellular volumes, etc.) are set by default.

## Introduction

The standard well-stirred, flow-limited PBPK differential equation describing the solute balance of organ i is deceptively simple [1]:


(1)

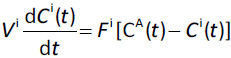



where *F*^i^ is the organ blood flow, *C*^A^(*t*) is the arterial blood concentration and *C*^i^(*t*) is the venous blood concentration leaving the organ and it is assumed that the free, unbound well-mixed tissue concentration is equal to the free concentration in the venous blood of the organ. All the complexity is in the parameter *V*^i^ which is the organ “volume of distribution” defined by:


(2)

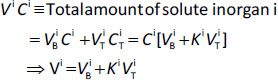



where *V*_B_^i^ and *V*_T_^i^ are the anatomic blood and extravascular organ volumes, respectively, *C*_T_^i^ is the tissue concentration and *K*^i^ (= *C*_T_^i^/*C*^i^) is the tissue/blood partition coefficient. The organ blood flow (*F*^i^) and the blood (*V*_B_^i^) and tissue (*V*_T_^i^) volumes can be accurately estimated from prior human physiological and anatomic measurements. However, *K*^i^, which depends on the specific physicochemical properties of the solute and varies from values of 0.2 or less to greater than 100, dominates the kinetics and is the major uncertainty and limitation of PBPK analysis.

In general, for an accurate PBPK analysis it is necessary to determine the *K*^i^ for the solute of interest from complex animal (usually rat) tissue measurements and then assume (hope) that the rat values can be extrapolated to the human. Ideally, one would like to be able to predict *K*^i^ just based on its chemical structure, allowing one to determine a drug’s detailed PK a priori. Unfortunately, for the majority of drugs that are weak acids or bases, it is not, as yet, possible to predict *K*^i^ accurately enough to meet this ideal. The current state of the art in predicting *K*^i^ is illustrated by the algorithm developed by Poulin and colleagues over many years of analysis [2]. About 50 % of the predictions are accurate to within a factor of 2, with about 15 % of the predictions off by a factor of greater than 3 fold. Although this level of accuracy is useful for, eg, predicting preliminary dosage levels for a trial drug, it severely limits the value of PBPK predictions because they are unlikely to be superior to those made using just a simple 1-compartment model based just on the standard pharmacokinetic (PK) measurements of clearance and volume of distribution.

**Figure 1.**
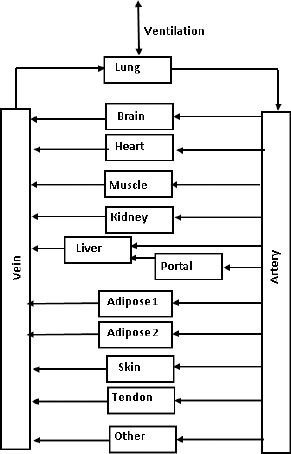
PBPK model used in PKQuest. The body is modeled by 12 tissue regions, arranged in parallel (except for the “portal” and “lung” tissue which are in series) connected by the “vein” and “artery” compartments. The tissue “portal” refers to the stomach, small and large intestine, spleen and pancreatic organs. The tissue parameters are listed in Table 1.

However, there are two major exceptions to this limitation: the highly lipid soluble (HLS) and the extracellular drugs (ECS). The HLS drugs (volatile anesthetics, propofol, cannabinol, etc.) have lipid/water partition coefficients (*P*_L/W)_ of 100 or greater and their K^i^ is dominated by the tissue fat fraction [1]. This means that one can predict the *K*^i^ just from in vitro measurements of *P*_L/W_ along with previous measurements of the fat fraction of all the tissues in the PBPK model (see Fig. 1). The ECS drugs, such as most antibiotics, cannot penetrate cells, and therefore are not subject to the intracellular binding that complicates the prediction of *K*^i^ for the weak bases and acids. The *K*^i^ of most ECS drugs is determined primarily by the volume and albumin concentration in the interstitial space and the albumin binding affinity. Thus, the ECS *K*^i^ can be predicted from prior measurements of tissue interstitial volumes and albumin concentrations and in vitro measurements of albumin binding affinity.

PKQuest is a freely distributed (www.pkquest.com), Java based, PK and PBPK program. The major effort in its design has been to provide a user interface that is both simple enough to be used by students as an adjunct in PK courses and general enough to be applicable to most PK and PBPK applications. Since its introduction in 2002, PKQuest has been applied to hundreds of solutes described in more than 11 publications [3-13]. Recently, a freely distributed textbook (“Computer assisted human pharmacokinetics”) has been developed that covers most PK topics and is closely integrated with PKQuest[1].

PKQuest implements the standard PBPK approach. The body is divided into 14 “tissue” compartments (Fig. 1), each of which is described by Eqs. (1) and (2) and three “tissue” parameters (*V*_T_^i^, *F*^i^, and *K*^i^). For a given solute input (oral, IV, etc.) the set of equations is solved numerically, and a plot of the time dependent solute concentration for each of the organs is plotted. Usually, of particular interest, is the time dependence of the concentration in the “vein” compartment.

In general, PBPK modeling requires input of a standard set of the 28 tissue volume (VTi) and blood flow (Fi) parameters. In PKQuest, a “standard human” set of these parameters has been refined by application of PKQuest to hundreds of solutes with varying properties. For example, the muscle blood flow was determined from the PK of D_2_O and the adipose blood flows from the PK of the volatile anesthetics (see below). These “standard human” values are listed in Table 1 (in PKQuest, the default units are minutes, liters and kilograms). Since these are the default background values, the user does not need to be concerned with supplying them. Of course, PKQuest allows these values to be varied in the more general case.

One of the novel features of PKQuest is that it is designed so that the PBPK modeling of HLS and ECS solutes can be run by entering just the minimum set of adjustable parameters (body fat fraction, *P*_L/W_, albumin binding, etc.) with all the other required human parameters as standard defaults. In addition to potentially predicting the PK of unknown solutes, this PBPK application is of heuristic value in, for example, teaching nurse anesthetists how the PK of volatile anesthetics depends on, e.g. respiratory rate. The following two sections will review the PK of these two solute classes along with clinical examples of the application of PKQuest to specific solutes.

## Highly lipid soluble solutes (HLS)

For an ideal HLS solute, the tissue/blood partition *K*^i^ is determined entirely by the lipid fraction of the tissue (*f*_L_^i^) and blood (*f*_L_^B^) and the lipid/water partition fraction (*P*_L/W_) [1]:


(3)

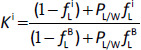



Equation (3) assumes that the blood HLS binding is due entirely to the blood lipid fraction (*f*_L_^B^). In humans, the directly measured *f*_L_^B^ is about 0.005 [12]. However, using this value often underestimates the blood HLS binding because there is additional plasma HLS binding, primarily by albumin. The procedure used in PKQuest is to input the in vitro measurement of the “free plasma fraction” if it is known and then use this value to estimate the apparent *f*_L_^B^. If it is not known, the default *f*_L_^B^ of 0.015 (Table 1) provides a rough starting estimate and it is then treated as an adjustable parameter.

The volatile anesthetics represent the ideal HLS because they are relatively inert, have negligible specific tissue binding and, in most cases, are not metabolized with their clearance determined solely by the respiration rate and blood/gas partition. Thus, their PK can be predicted using the HLS PBPK model along with in vitro measurements of oil/water, blood/water and air/water partition coefficients with no adjustable individual subject parameters. For the volatile anesthetics there are detailed measurements of the oil/water, air/water, blood/gas and various tissue partition coefficients (*K*^i^), all of which are qualitatively consistent with Eq. (3).

The PBPK parameter that dominates the PK of the HLS is the adipose tissue volume and blood flow. It is essential that the magnitude of the heterogeneity (if any) of adipose blood flow is included in the PBPK model. The time constant (*T*) for adipose tissue equilibration is described by (assuming a well-mixed, flow limited tissue):


(4)

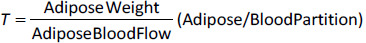



The units are kg for Adipose weight, and kg/min for blood flow while the adipose/blood partition is dimensionless.

For the volatile anesthetics *T* ranges from about 500 min to 3 days. At early times (several hours) before the lipid becomes saturated, the adipose tissue behaves like an infinite sink and the PK is not dependent on the heterogeneity. It is only at times that are long relative to *T* that the heterogeneity becomes apparent. Thus, in order to determine adipose perfusion heterogeneity it is necessary to have PK measurements that extend to days. Probably the best available set of measurements of this kind is the remarkable series of studies of the PK of the volatile anesthetics isoflurane, sevoflurane and desflurane by Eger and colleagues [14, 15]. They measured the ventilation rate and the inspired, mixed and end tidal gas concentration for 6 days following a 30 minute uptake in normal volunteers. Dr. Eger kindly provided this data which was then modeled using PKQuest to estimate the adipose perfusion heterogeneity. The analysis indicated that at least two equal volume adipose compartments with perfusion rates of 0.074 and 0.014 l/kg/min were needed to accurately model the long time data[10] and these are the default values in PKQuest (Table 1).

**Figure 2.**
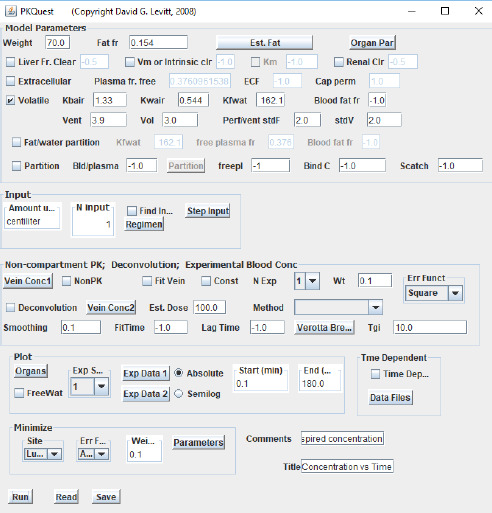
PKQuest interface. The top panel lists all the input required to specify the PBPK pharmacokinetics for isoflurane (see text for details).

Figure 2 shows the interactive PKQuest interface for isoflurane. The isoflurane kinetics are completely characterized by the entries in the top panel. The check box “Volatile” turns on the PBPK for volatile solutes (which are, presumably, also HLS). Setting *K*bair, *K*wair, *K*fwat characterizes isoflurane. The parameters Vent (alveolar ventilation) and Vol (alveolar volume) are the standard 70 kg human values. The entries in the other panels describe the details of the dose regimen and the variables that should be plotted in the output.

**Figure 3.**
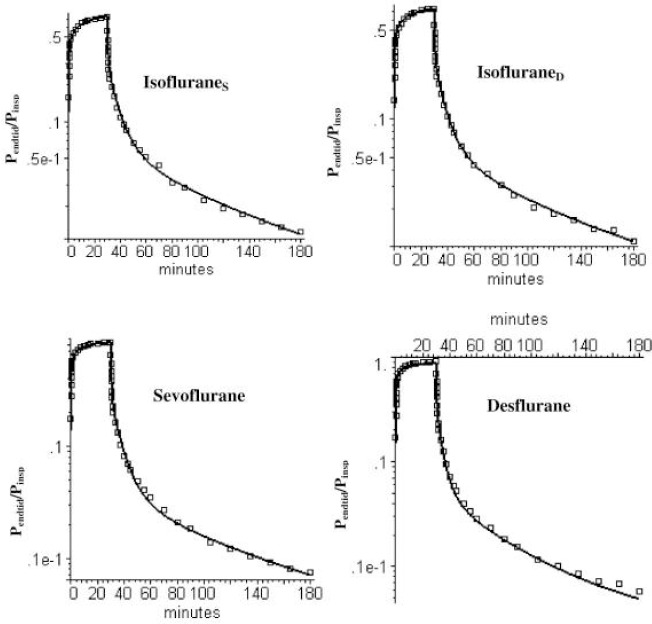
PKQuest PBPK predictions (black line) of short time pharmacokinetics of isoflurane, sevoflurane and desflurane using the identical basic PBPK model with two equal weight adipose organs with blood perfusions of 0.014 and 0.074 l/kg/min. Isoflurane_S_ and Isoflurane_D_ refer to two different sets of data.

**Figure 4.**
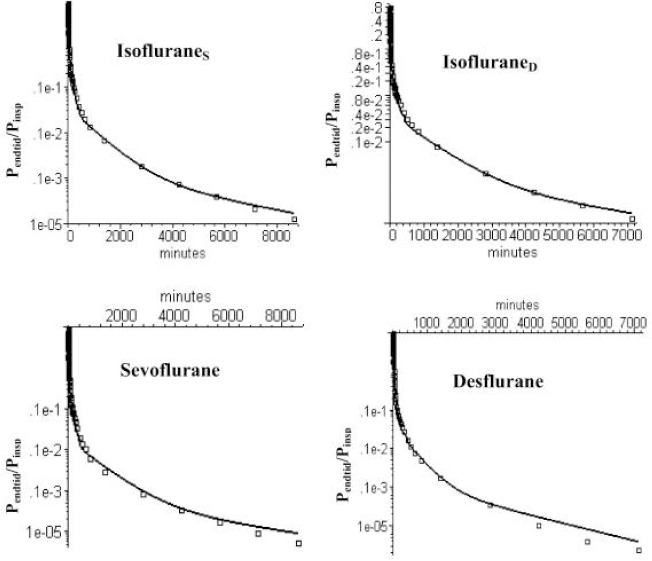
PKQuest PBPK predictions (black line) of long time pharmacokinetics of isoflurane, sevoflurane and desflurane using the identical basic PBPK model with two equal weight adipose organs with blood perfusions of 0.014 and 0.074 l/kg/min. Isoflurane_S_ and Isoflurane_D_ refer to two different sets of data.

Figures 3 and 4 show semi-log plots of the PKQuest PBPK prediction of the short and long time expired partial pressure (*P*_Expired_) relative to the *P*_Inspired_ during the 30 minute uptake period for isoflurane, sevoflurane and desflurane. It can be seen that, although these 3 anesthetics have significantly different properties (blood/air and oil/air partition), the human PK can be accurately fit using the same default PBPK model with no adjustable parameters. PBPK analysis is ideally suited for modeling the volatile anesthetics and has clear advantages over the 5 compartment mammillary model with 10 adjustable parameters that is classically used for these solutes [14, 15].

**Figure 5.**
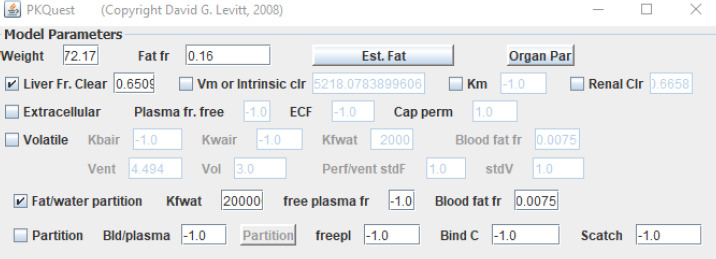
Top panel of the PKQuest interface for cannabinol (see text for details)

**Figure 6.**
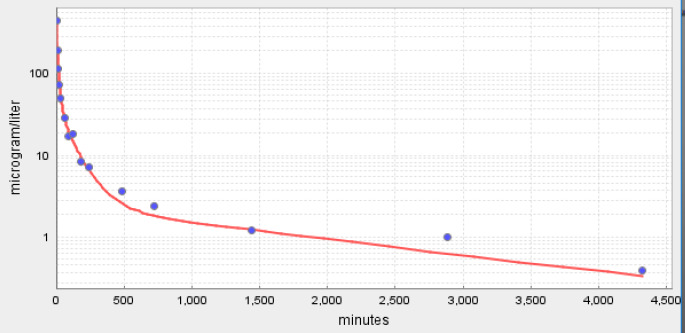
PKQuest PBPK predictions (red line) of pharmacokinetics of cannabinol

This same set of PBPK organ parameters is also applicable to non-volatile HLS. Figure 5 shows the PKQuest interface top panel for cannabinol. The “Fat/water partition” check box turns on the HLS option. Cannabinol has a very high oil/water partition of about 200,000 [10] (input in the “*K*fwat” box.). Only two adjustable parameters were required to provide a good fit to the PK data of Johansson et. al. [16]: the fractional liver clearance (0.651) and the blood fat fraction (0.0075). Figure 6 is the PKQuest output showing the PKQuest fit to this data.

**Figure 7.**
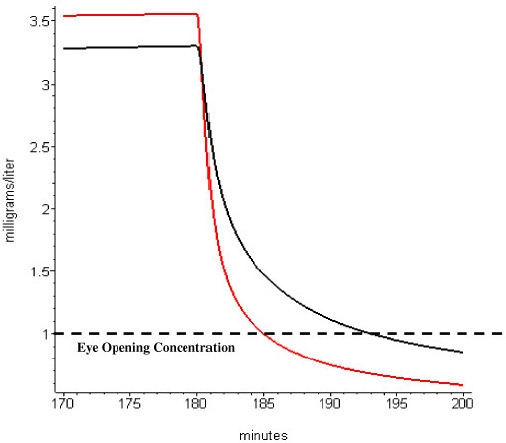
Plot of arterial blood propofol concentration in normal weight (black) and morbidly obese subjects (red) following a 180 minute propofol infusion. At a time of 180 minutes, the constant propofol infusion is terminated. The time for the arterial level to drop below the “eye opening concentration” is about 5 minutes for the obese and 13 minutes for the normal subjects

**Figure 8.**
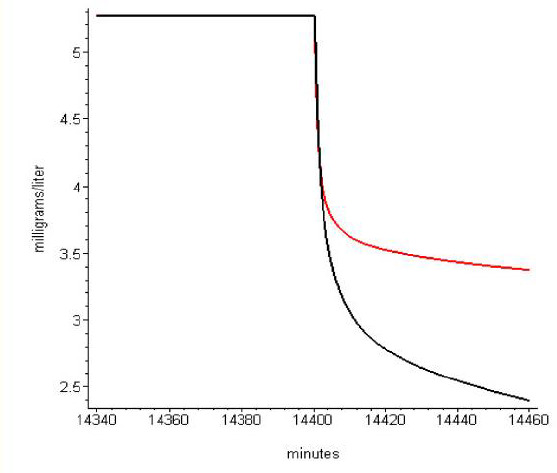
Comparison of arterial propofol concentration in normal (black) and obese (red) subjects during washout following a 10 day (14,400 minutes) constant infusion. The constant infusion rate has been adjusted so that the concentrations at the end of the 10 day period are identical for the normal and obese subjects.

An important clinical application of the PBPK HLS model is in predicting the PK of anesthetics in obese subjects. The prediction is obtained simply by changing the “Fat fr” entry in the PKQuest interface (Figs. 2 and 5). Figures 7 and 8 show two examples obtained from the PKQuest analysis of propofol [17]. Servin et. al. [18] had previously shown that the “eye opening” time following a 180 minute propofol infusion was shorter in obese subjects (10.3 min) versus normal weight subjects (18.4 min). Figure 7 shows that the PBPK model provides a similar prediction. Propofol is routinely used for long term sedation. Figure 8 shows the arterial concentration during the washout period following a 10 day constant infusion. It can be seen that in obese subjects (red) the washout is markedly slower with a greatly prolonged waking time. In determining the PK for the obese subjects, it is assumed that the standard human tissue parameters, including those for “Adipose1” and “Adipose2” (Table 1) can be extrapolated to the obese subjects. This is a major limitation because a careful evaluation of these parameters is not available for these subjects.

The extreme limit of HLS solutes are the persistent organic pollutants (POP), such as dioxins and polychlorinated biphenyls, which are characterized by human life times of several years and have lipid/water (*P*_L/W_) partition ranging from 10^5^ to greater than 10^7^ [12]. Because of these long lifetimes and the near impossibility of obtaining accurate experimental human PK data, POP modeling and prediction has become one of the most important PBPK applications. There is a common misperception that the long lifetimes results, primarily, from their very high lipid partition and resultant slow adipose tissue washout [19]. What is not commonly recognized is that, as described in eq. (3), for *P*_L/W_ greater than about 1000, *K*^Adipose^ reaches a maximum value equal to *f*_L_^Adipose^/*f*_L_^Blood^, and further increases in *P*_L/W_ do not increase it above this maximum value described by::


(5)

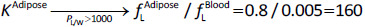



Substituting this relation for the maximum value of *K*^Adipose^ into eq. (4), the longest possible time constant for flow-limited adipose washout is:


(6)

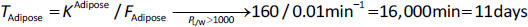



where *F*_Adipose_ is the adipose perfusion rate. Thus, high lipid partition can only account for washout time constants of about 11 days, much shorter than the year or more that is observed for many POPs.

One possible explanation is that Eq. (6) is incorrect because it assumed that the adipose/blood exchange is flow limited, while, as clearly shown by Levitt [12], it becomes diffusion limited for some POPs. This diffusion limitation arises because the high *P*_L/W_ of the POP produces such a low free water concentration that diffusion through the capillary wall becomes rate limiting. However, even taking account of this diffusion limitation, the adipose exchange time constant is at least 10 fold shorter than the POP lifetimes and cannot be responsible for the observed human POP persistence [12].

The explanation of this discrepancy between the adipose POP time constant and the whole body human time constant is simply that the PK of the POPs are limited by their extremely low rate of metabolism and excretion, not by the adipose/blood exchange. As the lipid partition increases, the free water concentration in the blood decreases and, presumably, the rate of liver metabolism decreases proportionally. If the metabolic time constant for excretion is long compared to the time constant for adipose and other tissue exchange, then at long times, one can ignore the PBPK model details and the plasma concentration can be described by a simple 1-compartment model characterized by its clearance (Cl) and volume of distribution (*V*):


(7)

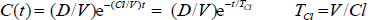



where *D* is the dose and *T*_Cl_ is the metabolic (ie, liver) time constant. Although the detailed PBPK model is required to describe the short time PK of the POPs, at long times the PK depends only on the liver clearance and is independent of the kinetics of solute exchange in the peripheral tissues.

**Figure 9.**
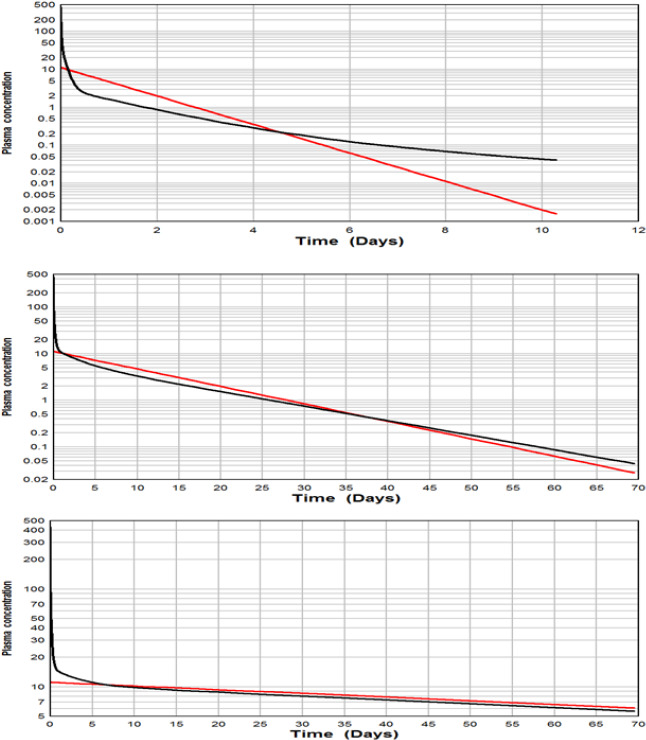
Comparison of 1-compartment (red) vs PBPK (black) model for HLS (eg, cannabinol) with an adipose time constant of about 11 days. As the metabolic time constant (ie, liver clearance) increases from 1.25 days (top), to 12.5 (middle) to 125 days (bottom), the PBPK model approaches the 1-compartment model at times longer than 11 days.

This is quantitatively illustrated using the PBPK cannabinol model described above [12]. Figure 9 shows a comparison of the plasma cannabinol concentration for the PBPK (black line) versus the 1-compartment model (red line) as the metabolic time constant (*T*_Cl_) increases from 1.25 days (top panel) to 125 days (bottom panel). It can be seen (bottom panel) that when the metabolic excretion rate becomes rate limiting (*T*_Cl_ = 125 days >> *T*_Adipose_ =11 days), the one compartment model provides a good prediction of the PK after the initial ≈11 day transient period when the adipose tissue exchange is limiting.

## Extracellular solutes (ECS)

Because the ECS, by definition, do not enter cells, they are not subject to the poorly characterized intracellular binding that confounds predictions of the *K*^i^ for the typical weak acid or base. Since the ECS solutes are confined to the plasma and interstitial space, the basic PBPK equations described above (eqs. (1) and (2)) are modified as follows:


(8)

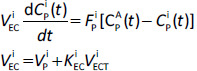



where *F*_P_^i^ is the organ plasma flow, *C*_P_^A^ is the plasma arterial concentration, and *C*_P_^i^ is the venous plasma concentration. The ECS volume of distribution (*V*_EC_^i^) is defined in terms of the anatomical plasma (*V*_P_^i^) and interstitial (*V*_ECT_^i^) volumes and the ECS interstitial/plasma partition coefficient (*K*_EC_^i^). Because both the plasma and interstitial ECS binding is dominated by the plasma and interstitial albumin, it can be shown [7] that the ECS partition coefficient (*K*_EC_^i^) can be described by:


(9)

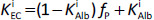



where *K*_Alb_^i^ is ratio of interstitial/plasma albumin concentration in organ i and *f*_P_ is the fraction of the ECS solute that is free (unbound) in plasma and characterizes the ECS albumin binding affinity. If *f*_p_ = 1 (no albumin binding), *K*_EC_^i^ = 1. If *f*_p_ = 0 (very high affinity binding), *K*_EC_^i^ = *K*_Alb_^i^.

Levitt [7] has carried out a detailed review of the literature and determined an optimal set of values for *K*_Alb_^i^ (the ratio of interstitial/plasma albumin concentration) and the fraction of each organ that is extracellular (ECF fraction) with *V*_ECT_^i^ (the anatomic interstitial volume for each organ i) equal to the ECF fraction times the total organ volume. These parameters are listed in Table 1 and are the default parameters in PKQuest. Using these predetermined values, the only parameter that is solute specific is *f*_p_ (the free solute fraction in plasma) and this can be determined from in vitro measurements. Thus, all of the parameters in Eqs. (8) and (9) can be determined a priori, providing a complete description of the ECS PBPK.

**Figure 10.**
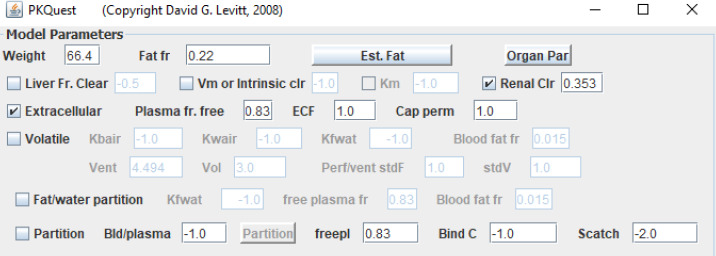
Top panel of PKQuest interface showing the parameters that characterize amoxicillin PBPK pharmacokinetics (see text for details).

**Figure 11.**
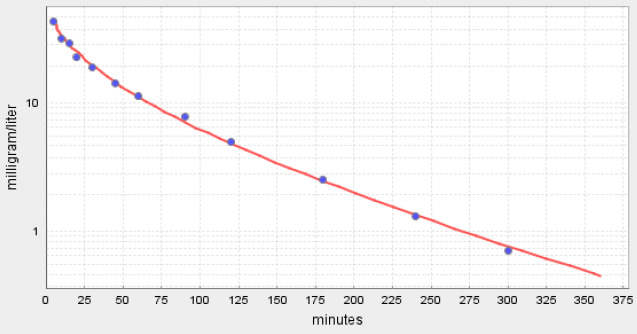
Semi-log plot of comparison of experimental antecubital amoxicillin concentration (blue circles) and PKQuest PBPK predicted antecubital concentration (red line) following a bolus IV input.

For most ECS, there is only one adjustable parameter, the clearance (usually renal). Figure 10 shows the top panel of the PKQuest interface for amoxicillin. The “Extracellular” check box turns on the ECS PBPK model. The “freepl” is the fraction free in plasma (= *f*_P_), which characterizes both the plasma and interstitial albumin binding. The only adjustable parameter is the “Renal Clr” which is set to 0.353 (the fraction of renal plasma cleared). Note also that the “Cap perm” is set to 1.0, indicating infinite capillary permeability (see below). Figure 11 shows the good fit of the PKQuest PBPK prediction to the experimental data of Arancibia et. al.[20] following a bolus IV input. For some solutes the plasma albumin binding (= freepl) is poorly characterized or variable, and one treats freepl as another adjustable model parameter. Using this identical PBPK model, similarly good fits were obtained to the experimental PK measurements for the ECS solutes EDTA, DTPA, morphine-6-glucuronide, morphine-3-glucuronide, mannitol and for the β-lactam antibiotics amoxicillin, piperacillin and cefatrizine [7].

For all the solutes discussed above, the standard PBPK “flow-limited, well-stirred” model has been assumed for the tissue PK. This assumes that the free, unbound concentration in the tissue space is uniform for the entire tissue and is equal to the free plasma concentration that leaves the organ in the vein. This assumes, in essence, that the capillary permeability is infinite. This assumption may be incorrect because the ECS solutes cannot penetrate cell membranes and must traverse the capillary wall through the potentially limiting interendothelial slits [21].

**Figure 12.**
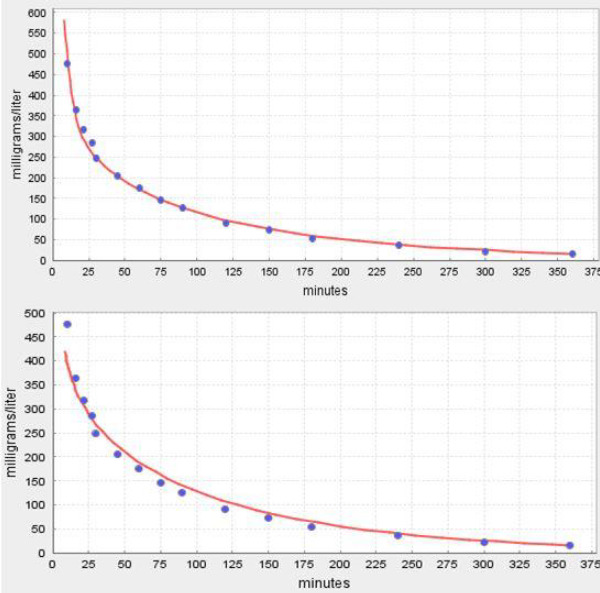
PKQuest PBPK pharmacokinetics (red line) for inulin following a 5 minute constant IV infusion. The top panel is with a capillary permeability limitation (fclr^muscle^ = 0.45) and the bottom panel is for an infinite permeability (fclr^muscle^ = 1).

There is no question that some ECS solutes have a capillary permeability limitation. The classical example is inulin, whose capillary permeability has been determined in animal models using a variety of experimental approaches [21]. PKQuest has been modified to allow for a capillary permeability limitation characterized by the parameter “flcr”, the fraction of plasma solute that equilibrates with the interstitial space in one pass through the capillary. It is defined by:


(10)

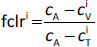



where *c*_A_, *c*_v_^i^ and *c*_t_^i^ are the free unbound concentrations in the artery, vein and tissue, respectively. If the capillary permeability is infinite, *c*_v_^i^ = *c*_t_^i^ and fclr = 1. If the permeability is 0, *c*_A_ =*c*_v_ and fclr = 0. In PKQuest, the input parameter “Cap perm” (Fig. 9) is the fclrmuscle. The flcr of the other tissues are then set equal to proportional values determined from a literature review [7]. Figure 12 shows a comparison of the permeability limited (top panel) versus infinite permeability (bottom panel) PKQuest PBPK fit to the inulin data of Odeh et al. [22]. It can be seen that although the permeability limitation only affects the early time data and is a relatively small effect, the addition of the limitation clearly improves the fit. The best fit was obtained with the fclrmuslc of 0.45 which corresponds to a capillary permeability (PS, eq. (11)) of skeletal muscle of 0.61 ml/min/100 gm, similar to that determined directly in animal studies [21]. This PBPK approach represents a new way to study capillary permeability and is the first human measurement of muscle capillary permeability for these solutes.

It had not been previously recognized that, as the albumin binding affinity increases, the capillary permeability should decrease. One can show that the parameter fclr^i^ is described by the relation [3,4,7]:


(11)

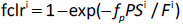



where *f*_p_ is the fraction free in plasma, *P* is the capillary permeability of the free, unbound solute, and *S*^i^ and *F*^i^ are the capillary surface area and flow rate for tissue i. As *P* approaches infinity, fclr approaches 1 and the solute becomes flow limited. It can be seen that solutes that have a high “intrinsic” *P* if they are unbound, may become capillary limited as the albumin binding affinity increases and fp approaches zero.

**Figure 13.**
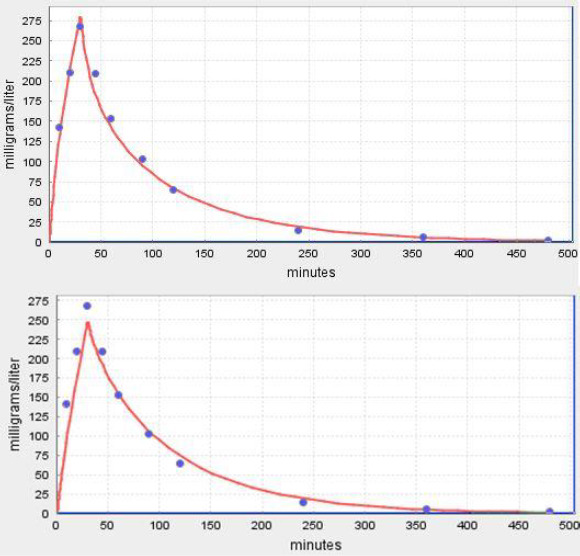
PKQuest PBPK pharmacokinetics (red line) for dicloxacillin following a 30 minute constant IV infusion. The top panel is with a capillary permeability limitation (fclr^muscle^ = 0.3) and the bottom panel is for an infinite permeability (fclr^muscle^ = 1).

This *f*_p_ dependence of the capillary permeability was tested using the experimental PK of a series of β-lactam antibiotics with *f*_p_ varying from 0.8 for amoxicillin, to 0.52 for piperacillin, to 0.07 for flucloxacillin and 0.03 for dicloxacillin [7]. The quality of the PKQuest PBPK fits to the experimental data with and without a capillary permeability limitation was compared. For flucloxacillin and dicloxacillin, the two solutes with the highest albumin binding, the fit clearly improved when a permeability limitation was introduced. Figure 13 shows a comparison of the PKQuest output for dicloxacillin with a permeability limitation (top panel, fclr^muscle^ = 0.3) versus infinite permeability (bottom panel, fclr^muscle^ = 1). Again it can be seen that although the permeability limitation only affects the early time data and is a relatively small effect, the addition of the limitation clearly improves the fit. The estimated PBPK human PS values are similar to those of EDTA and mannitol, ECS solutes of similar size, measured directly in the cat [7].

The possibility of a capillary permeability limitation complicates the PK of the ECS solutes. As shown above, for solutes the size of the β-lactam antibiotics and *f*_p_ greater than 0.3, it can be assumed that the permeability is infinite. For larger solutes (eg, inulin) or higher albumin binding affinity (eg, dicloxacillin), there may be significant permeability limitation that can be modeled by treating the PKQuest capillary permeability parameter “fclr” as an additional adjustable parameter.

## Conclusion

It is clear from the above discussion that the HLS and ECS represent the ideal solutes for PBPK analysis. The PK of these solutes can usually be accurately predicted using only zero (volatile anesthetics) or one adjustable parameter (the clearance). Not only can one predict the blood or plasma PK, but having so few adjustable parameters increases ones confidence in the PBPK model, allowing the determination of the time dependent concentration in the different tissues and how the PK varies with, eg, exercise or body fat fraction. Unfortunately, the HLS and ECS solutes classes are limited, with the examples discussed above nearly exhausting the important drug classes. In contrast, for the much more common weak acid or base solutes, an accurate PBPK model requires detailed animal experimental measurement of the individual organ *K*^i^, and then an uncertain extrapolation to the human. These factors have significantly limited the confidence in the use of PBPK models for, eg, toxicological applications [23]. It does not seem to be widely recognized that the HLS and ECS solutes are important exceptions to the usual problems associated with PBPK analysis and it is hoped that this discussion will remedy this.

This discussion has focused on only one aspect of PKQuest: the PBPK analysis of the HLS and ECS solutes. PKQuest also offers several other novel PBPK features, including: 1) the first incorporation of the use of antecubital vein sampling in a PBPK model [9]; and 2) the first ethanol PBPK model with a rigorous definition of the non-linear bioavailability [6]. In addition to the PBPK module, PKQuest also has a number of other PK features. There is major emphasis on deconvolution which is a powerful and underutilized modeling approach. There are 6 different deconvolution routines available, each with its strengths and limitations. One novel deconvolution application is a general approach to determine the intestinal permeability during normal human drug absorption, with application of this approach to 90 different drugs [13]. All of these features are discussed with detailed worked examples in the freely distributed textbook “Computer assisted human pharmacokinetics” [1]. It is hoped that the interested reader will download the free software and textbook from www.pkquest.com and try out these and other PKQuest PK features.

## Figures and Tables

**Table 1.** Organ PBPK parameters for the standard 20 % fat, 70 kg human. The organ “portal” refers to all the organs drained by the portal vein (eg, GI tract, spleen, pancreas). The organs “tendon” and “other” represent poorly characterized, low flow connective tissues. The adipose and adipose 2 organ weights (assumed equal) are adjusted for the individual’s body fat fraction. The ECF fraction is the fraction of the non-lipid organ weight that is extracellular. K_Alb_ is the ratio of the albumin concentration in the EDTA interstitial space relative to the plasma albumin.

Organ	Weight (kg)	Perfusion (l/min/kg)	Lipid Fraction	ECF fraction	*K*_Alb_
vein	4.29	1.0	0.015	0.595	NA
artery	1.21	0.0	0.015	0.595	NA
liver	1.8	0.25	0.02	0.23	0.5
portal	1.5	0.75	0.016	0.3	0.35
kidney	0.31	4.0	0.0136	0.165	0.35
brain	1.4	0.56	0.0176	0	0.1
heart	0.33	0.8	0.0136	0.264	0.5
muscle	26.0	0.0225	0.0136	0.15	0.5
skin	2.6	0.1	0.0136	0.6	0.25
lung	0.536	-1.0	0.0136	0.2	0.35
tendon	3.0	0.01	0.0136	1	0.25
other	5.522	0.02	0.0136	0.2	0.25
adipose	8.758	0.074	0.8	1	0.35
adipose 2	8.758	0.01408	0.8	1	0.35
Bone	4.0	0.0	0	0	0
